# Rejuvenating Hyaline Cartilage with Senescence‐Targeting Si‐ADAM19 Delivery for Osteoarthritis Therapy

**DOI:** 10.1002/advs.202414419

**Published:** 2025-02-10

**Authors:** Jiasheng Wang, Peng Guo, Dongmei Wu, Junzhi Yi, Qi Jiang, Jiajie Hu, Hongwei Ouyang

**Affiliations:** ^1^ Department of Sports Medicine of the Second Affiliated Hospital and Liangzhu Laboratory Zhejiang University School of Medicine Hangzhou 310058 China; ^2^ Dr. Li Dak Sum & Yip Yio Chin Center for Stem Cells and Regenerative Medicine Zhejiang University School of Medicine Hangzhou 310058 China; ^3^ Zhejiang University‐University of Edinburgh Institute Zhejiang University School of Medicine Haining 310058 China; ^4^ China Orthopedic Regenerative Medicine Group (CORMed) Hangzhou China

**Keywords:** a disintegrin and metalloproteinase 19 (ADAM19), cartilage regeneration, cellular senescence, osteoarthritis(OA), rejuvenating, siRNA delivery

## Abstract

Osteoarthritis (OA) is one of the most common joint degenerative diseases without effective treatment, whose pathology is related to the local accumulation of senescent cells (SnCs). However, existing SnCs‐scavenging drugs “senolytics” may lead to the exhaustion of stem and progenitor cells, impairing chondrocyte proliferation and cartilage regeneration. Here, ADAM19, a kind of endopeptidases from the ADAM (a disintegrin and metalloproteinase) family, is identified as a novel target for senescent chondrocyte rejuvenation. ADAM19 is elevated in senescent chondrocytes in both mice and human osteoarthritic joints, as well as in cellular senescence model in vitro. ADAM19 knockdown not only significantly attenuated senescent phenotype of chondrocytes, but also promoted cell proliferation and extracellular matrix synthesis. RNA sequencing revealed ADAM19 may regulate chondrocyte senescence mainly through the PI3K/AKT signal axis. In addition, a senescence‐targeting small interfering RNA (siRNA) delivery system is developed for in vivo delivery of therapeutic siRNA. The complex selectively released ADAM19 siRNA in SnCs and performed high silencing effect on target gene. Furthermore, intra‐articular (IA) injection of the complex once every two weeks in OA mice effectively reduced SnCs accumulation and promoted hyaline cartilage regeneration. This study provides a promising strategy for the development of regenerative RNA interference therapy.

## Graphical abstract

ADAM19 is identified as a novel target for senescent chondrocyte rejuvenation. Knockdown of ADAM19 results in the attenuation of cellular senescence and the formation of proliferative extracellular matrix‐synthesizing chondrocytes. senHMSN_si‐ADAM19 can target deliver therapeutic siRNA into senescent cells and rejuvenate hyaline cartilage in experimental OA mice. This work introduces a promising idea for OA therapeutic agents.

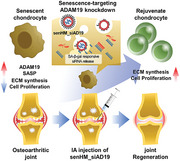

## 1 Introduction

Osteoarthritis (OA) is the most prevalent degenerative joint disease in the world and a leading cause of lower limb disability among elderly people, which imposes an enormous socioeconomic costs on healthcare systems and lacks effective therapeutics.^[^ ^1^ ^]^ OA is thought to be the result of a combination of multi‐factors, however, aging is the most leading risk factor.^[^ ^2^ ^]^ As one of the hallmarks of aging, cellular senescence is a special form of cell fate characterized by irreversible cell cycle arrest induced by increasing age or continuous stress stimuli.^[^ ^3^ ^]^ Senescent cells (SnCs) are characterized by an abnormal increase of lysosomal senescence‐associated β‐galactosidase (SA‐β‐gal) activity and the release of pro‐inflammatory molecules called the senescence‐associated secretory phenotype (SASP), which deteriorate tissue microenvironment and cause age‐related pathologies.^[^ ^4^ ^]^ Researches have shown that SnCs were accumulated during OA progression^[^ ^5^ ^]^ and local transplantation of SnCs into the knee joint induces an OA‐like phenotype.^[^ ^6^ ^]^ Elimination of damaged or senescent chondrocytes could effectively alleviate the progression of OA.^[^ ^7^ ^]^ Therefore, the accumulation of SnCs in the joint could be one of the key factors initiating OA and therapies targeting SnCs might be an attractive treatment for OA.

Existing strategies targeting SnCs have been proposed: senolytics induce apoptosis in SnCs, whereas senomorphics inhibit or neutralize SASP.^[^ ^8^ ^]^ Senolytics have shown a range of promising results, however, as an agent based on recognition of apoptosis‐resistance of SnCs, long‐term use may cause undesirable side effects.^[^ ^9^ ^]^ Importantly, removing senescent stem cells leads to the exhaustion of stem cell and progenitor cell pools, further impairing cell proliferation and tissue regeneration.^[^ ^10^ ^]^ For senomorphics, matrix metalloproteinase 13 (MMP13) inhibitors have been shown to be effective in reducing the severity of post‐traumatic OA in mice.^[^ ^11^ ^]^ Other SASP regulators such as microRNAs have also shown potent SASP regulation and cartilage protection.^[^ ^12^ ^]^ Although senomorphics could intervene SASP and reduce the impact of pro‐inflammatory factors on healthy chondrocytes, the potential problem of senomorphics is their failure to modify SnCs, which means the drug cannot effectively reverse the impaired cell proliferation and tissue regeneration mediated by the accumulation of SnCs. More importantly, cartilage has limited regenerative capacity because of the poor replicative ability of chondrocytes.^[^ ^8^ ^]^ Therefore, it is of critical importance to explore novel rejuvenate targets and effective delivery systems for replenishing the osteoarthritic cartilage with robust regenerative chondrocytes.

A Disintegrin and Metalloproteinases (ADAMs) are a family of transmembrane metalloproteinases, which have endopeptidase activity and can cleave extracellular matrix proteins.^[^ ^13^ ^]^ Family member ADAM19, also called meltrin β, is mainly distributed in peripheral nervous system, skeletal muscle, bone, and heart tissues and performs important biological functions in embryonic development.^[^ ^14^ ^]^ Under pathological conditions, ADAM19 is over‐expressed in a variety of tumors and closely correlated to tumor growth and metastasis.^[^ ^15^ ^]^ Abnormally high expression of ADAM19 has also been reported to be associated with inflammation and fibrosis of the lung and kidney.^[^ ^16^ ^]^ In addition, ADAM19 promotes obesity and enhances insulin resistance, which involves in the pathogenesis of type 2 diabetes.^[^ ^17^ ^]^ These findings underscore the pivotal role of ADAM19 in the extracellular matrix remodeling, cell proliferation, tissue inflammation, and obesity‐related metabolic diseases. However, whether ADAM19 is involved in the cellular senescence and progression of OA or other age‐related diseases remains unclear.

Although multiple regulatory genes of senescence have been discovered, how to selectively regulate the expression of these genes in SnCs to achieve therapeutic effects remains a great challenge. Small interfering RNA (siRNA)‐based nanotherapies have emerged as a promising therapeutic platform for OA treatment.^[^ ^18^ ^]^ However, the instability of siRNAs makes them can be easily removed from the synovial fluid and subjected to nuclease degradation within a few hours.^[^ ^19^ ^]^ Moreover, cartilage is a non‐vascular tissue with dense extracellular matrix, leading to the delivery of therapeutic siRNA to chondrocytes is still a challenge.^[^ ^20^ ^]^ Although nanoplatforms have been developed to effectively deliver nucleic acid drugs, they lack specificity for target cells or tissues. Therefore, delivering sufficient and intact siRNA to senescent chondrocytes still facing crucial problems.

In this work, we identified ADAM19 as a novel target for senescent chondrocyte rejuvenation and constructed a senescence‐responsive nucleic acid delivery system for targeted release of therapeutic siRNA in SnCs. Our findings introduced a promising idea for the development of therapeutic agents for OA treatment.

## 2 Results

### 2.1 ADAM19‐Positive Senescent Chondrocytes Accumulated in both Mice and Human Osteoarthritic Joints

**Figure 1:**
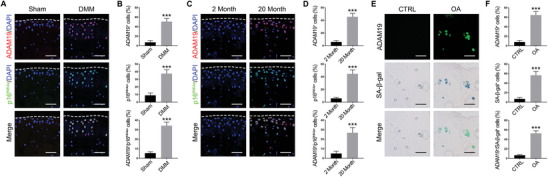
The expression of ADAM19 in post‐traumatic and naturally occurring OA mice and OA patients. A, B) Representative immunofluorescence images of ADAM19 (red) and p16^INK4a^ (green) in post‐traumatic OA mouse articular cartilage (A) and quantification of ADAM19, p16^INK4a^, and ADAM19/p16^INK4a^‐double positive cells (B). *n* = 3 mice for sham and *n* = 5 for DMM surgery. C, D) Representative immunofluorescence images of ADAM19 (red) and p16^INK4a^ (green) in naturally occurring OA mouse (C) and quantification of ADAM19, p16^INK4a^, and ADAM19/p16^INK4a^‐double positive cells (D). *n* = 3 mice for 2‐month‐old and *n* = 5 for 20‐month. E, F) Representative immunofluorescence of ADAM19 (green) and SA‐β‐gal (blue) co‐staining in human cartilage (E) and quantification of ADAM19, SA‐β‐gal, and ADAM19/SA‐β‐gal‐double positive cells (F). *n* = 3 donors for control and *n* = 5 for OA. Scale bar, 50 µm. Dotted lines indicate the cartilage surface. Data are presented as means ± SD. ***p < 0.001.

To detect whether ADAM19 is expressed in SnCs under OA pathologic conditions, we first performed destabilization of the medial meniscus (DMM) surgery to induce post‐traumatic OA in mice and found the number of ADAM19‐positive cells and p16^INK4a^‐positive SnCs were markedly increased in the osteoarthritic cartilage rather than in the sham joint (**Figure** **1**A,B). Furthermore, ADAM19‐or p16^INK4a^‐expressing cells were also increased in the articular cartilage in aged (20‐month‐old) mice (Figure 1C,D). Notably, the number of ADAM19‐and p16^INK4a^‐double positive SnCs in DMM (34.2 ± 3.70%) or aged (26.8 ± 5.50%) mice was significantly higher than that in sham (5.4 ± 1.52%) or adult (5.0 ± 2.55%) mice (Figure 1B,D). Similar to the murine results, ADAM19 was highly expressed and the number of ADAM19/SA‐β‐gal or ADAM19/p16^INK4a^ co‐stained SnCs were both increased in the knee joint cartilage of OA patients (Figure 1E,F; Figure S1, Supporting Information). The location of ADAM19‐expressing cells was highly coincident with SA‐β‐gal‐positive cells in the OA samples, in which 52.6 ± 5.41% of cells were ADAM19 and SA‐β‐gal double positive SnCs, while only 6.6 ± 1.52% in the normal control (Figure 1F). These results indicated that ADAM19‐positive senescent chondrocytes accumulated in osteoarthritic joints.

### 2.2 Chondrocyte Senescence Elevates the Expression of ADAM19

**Figure 2:**
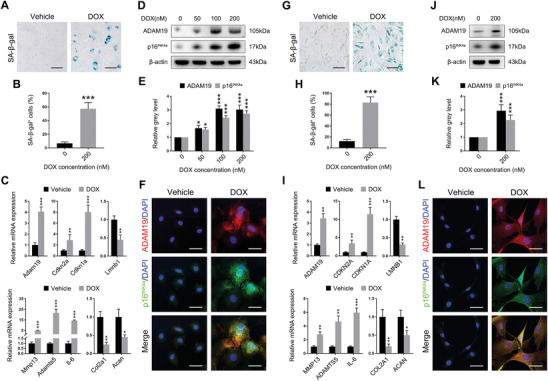
The expression of ADAM19 in mouse and human chondrocyte. A, B) Representative images of SA‐β‐gal staining of mouse chondrocytes treated with 200n M DOX (A) and quantification of SA‐β‐gal‐positive cells (B). Scale bar, 30 µm. C) Transcriptional levels of target genes in mouse chondrocytes treated with 200n M DOX. D, E) The protein level of ADAM19 and p16^INK4a^ in mouse chondrocytes treated with different concentrations of DOX (D) and relative grey level of ADAM19 and p16^INK4a^ protein bands normalized to β‐actin (E). F) Representative immunofluorescence images of ADAM19 (red) and p16^INK4a^ (green) in mouse chondrocytes treated with 200n M DOX. Scale bar, 50 µm. G, H) Representative images of SA‐β‐gal staining of human chondrocytes treated with 200n M DOX (G) and quantification of SA‐β‐gal‐positive cells (H). Scale bar, 30 µm. I) Transcriptional levels of target genes in human chondrocytes treated with 200 nM DOX. J, K) The protein level of ADAM19 and p16^INK4a^ in human chondrocytes treated with DOX (J) and relative grey level of ADAM19 and p16^INK4a^ protein bands normalized to β‐actin (K). L) Representative immunofluorescence images of ADAM19 (red) and p16^INK4a^ (green) in human chondrocytes treated with 200n M DOX. Scale bar, 50 µm. Data are presented as means ± SD of at least 3 independent experiments. *p < 0.05, **p < 0.01, ***p < 0.001.

We next examined whether ADAM19 was dysregulated in senescent chondrocytes in vitro. The DNA‐damaging chemical agents, such as doxorubicin (DOX), has been proved to induce cellular senescence or senescence like states.^[^ ^12^, ^21^ ^]^ DOX treatment caused robust cellular senescence in primary cultured mouse chondrocytes, as indicated by the increasing SA‐β‐gal activity and p16^INK4a^ expression (**Figure** **2**A–F). Chondrocytes treated with DOX exhibited multiple senescent phenotypes, such as induction of Cdkn2a and Cdkn1a, and the transcriptional loss of lamin B1 (Lmnb1). Chondrocyte senescence also promoted the expression of ADAM19, matrix degradative enzymes such as Mmp13, Adamts5, and suppressed extracellular matrix synthesis (Figure 2C). Western blot verified DOX elevated ADAM19 and p16^INK4a^ protein expression in mouse primary chondrocytes in a dose‐dependent manner (Figure 2D,E). Notably, immunofluorescence confirmed the co‐expression of ADAM19 in p16^INK4a^‐positive cells in mouse chondrocytes after DOX treatment (Figure 2F). Consistent with the murine results, DOX significantly up‐regulated ADAM19 expression in human primary chondrocytes, along with classic cellular senescence markers accumulation and pro‐inflammatory SASP transcription. Furthermore, the p16^INK4a^‐expressing cells in the DOX treated group were co‐stained with ADAM19 (Figure 2G–L). Other stimuli that elicit cellular senescence, such as ionizing radiation (IR), also prominently increased ADAM19 expression in both mouse and human chondrocyte (Figure S2, Supporting Information). Together, these data suggested that ADAM19 expression was elevated in both mouse and human senescent chondrocyte and ADAM19 might be a therapeutic target for cellular senescence.

### 2.3 ADAM19 Knockdown Attenuates Cellular Senescence and Promotes Regenerative Potential of Chondrocytes

**Figure 3:**
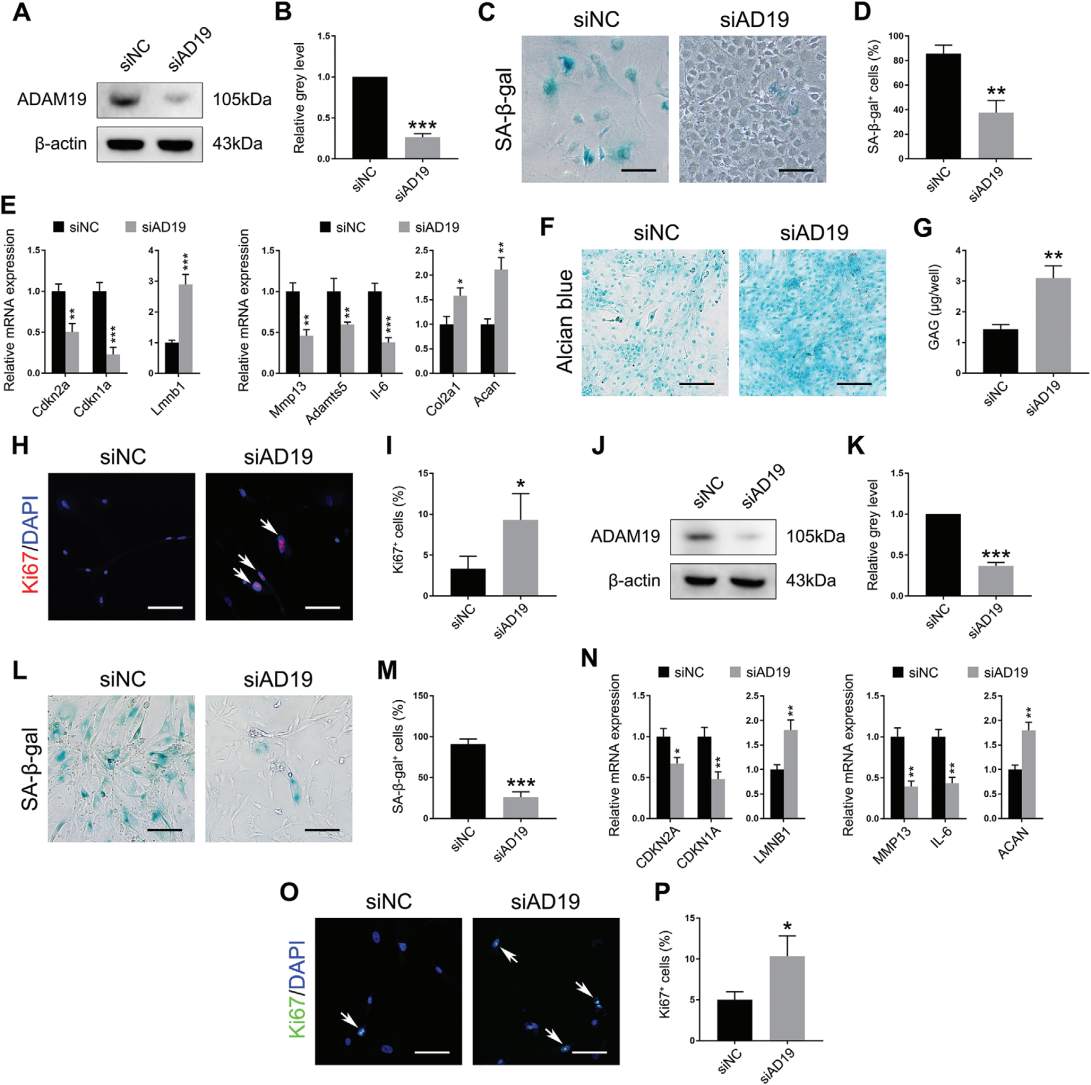
The effect of ADAM19 knockdown on chondrocyte senescence. A, B) The protein level of ADAM19 in senescent mouse chondrocytes treated with si‐NC (siNC) or si‐ADAM19 (siAD19) (A) and relative grey level of ADAM19 protein band normalized to β‐actin (B). C, D) Representative images of SA‐β‐gal staining of senescent mouse chondrocytes (C) and quantification of SA‐β‐gal‐positive cells (D). Scale bar, 30 µm. E) Transcriptional levels of target genes in senescent mouse chondrocytes. (F, G) Representative images of Alcian blue staining of senescent mouse chondrocytes F) and quantification of glycosaminoglycan expression using DMMB assays G). Scale bar, 200 µm. H, I) Representative immunofluorescence images of Ki‐67 (red) of senescent mouse chondrocytes (H) and quantification of Ki‐67‐positive cells (I). Scale bar, 50 µm. J, K) The protein level of ADAM19 in senescent human chondrocytes (J) and relative grey level of ADAM19 protein band normalized to β‐actin (K). L, M) Representative images of SA‐β‐gal staining of senescent human chondrocytes (L) and quantification of SA‐β‐gal‐positive cells (M). Scale bar, 30 µm. N) Transcriptional levels of target genes in senescent human chondrocytes. O, P) Representative immunofluorescence images of Ki‐67 (red) of senescent human chondrocytes (O) and quantification of Ki‐67‐positive cells (P). Scale bar, 50 µm. Data are presented as means ± SD of at least 3 independent experiments. *p < 0.05, **p < 0.01, ***p < 0.001.

To explore the pathophysiological role of up‐regulated ADAM19 in senescent chondrocytes, we used siRNA to knockdown ADAM19 in chondrocytes and the non‐targeting siRNA as the negative control (si‐NC). ADAM19 siRNA (si‐ADAM19) could effectively inhibit the ADAM19 protein in senescent mouse (**Figure** **3**A,B) and human primary chondrocytes (Figure 3J,K). The number of SA‐β‐gal‐positive SnCs transfected with si‐ADAM19 was significantly decreased compared with si‐NC (Figure 3C,D). ADAM19 knockdown inhibited cellular senescence markers, pro‐inflammatory SASP expression, and restored lmnb1 in senescent mouse chondrocyte (Figure 2E). Similarly, si‐ADAM19 decreased SA‐β‐gal positivity and ameliorated multiple senescent phenotypes in human senescent chondrocyte (Figure 3L–N). Next, we attempted to explore the therapeutic potential of si‐ADAM19 as a modulator of senescent chondrocyte regenerative phenotype. Adult chondrocytes have poor proliferative ability and are extremely difficult to repair once damaged.^[^ ^22^ ^]^ Previous senotherapies could attenuate chondrocyte senescence but hardly promote cartilage regeneration. Surprisingly, si‐ADAM19 treated senescent mouse chondrocytes showed higher expression of cartilage matrix components (Figure 3E) and deeper Alcian blue staining with higher glycosaminoglycan content compared with si‐NC (Figure 3F,G). Furthermore, ADAM19 knockdown in senescent chondrocytes presented more ki‐67‐positive proliferating cells (Figure 3H,I). human senescent chondrocyte exposed to si‐ADAM19 also exhibited a robust regenerative phenotype (Figure 3N–P). Collectively, these results suggested that si‐ADAM19 not only could alleviate chondrocyte senescence, but also promote cell proliferation and matrix synthesis. ADAM19 knockdown might be a valid strategy to rejuvenate hyaline cartilage.

### 2.4 ADAM19 Regulates Chondrocyte Senescence and SASP Expression Mainly through PI3K/AKT Signaling Pathway

**Figure 4:**
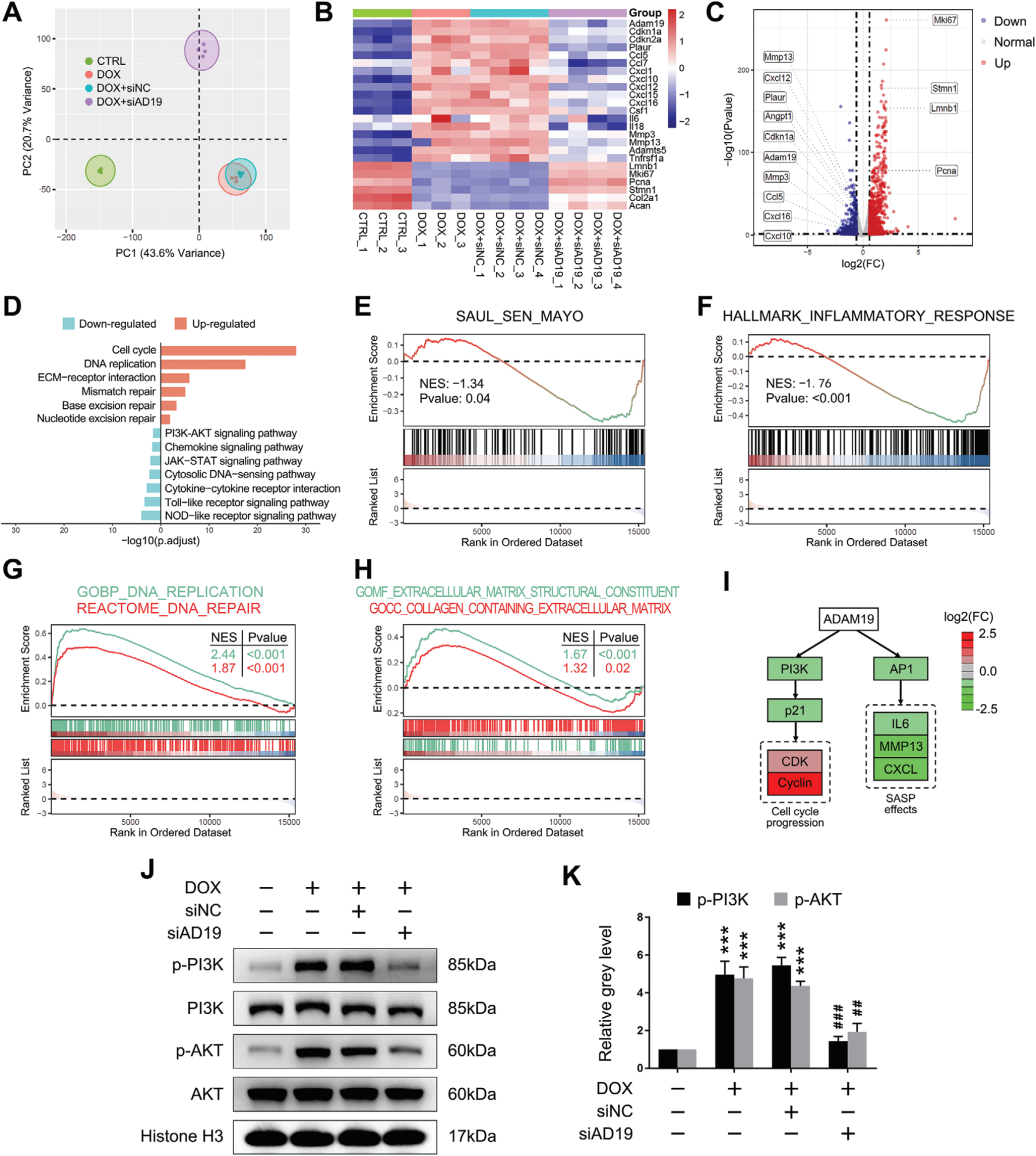
The gene expression profile of ADAM19 knockdown on chondrocyte senescence. A) PCA clustering of vehicle treated (CTRL), DOX treated (DOX), DOX treated and si‐NC (DOX+siNC) or si‐ADAM19 (DOX+siAD19) transfected of mouse chondrocytes. *n* = 3 for CTRL and DOX, *n* = 4 for DOX+siNC and DOX+siAD19. B) Heatmap of the selected differentially expressed genes (DEGs) between the groups. C) Volcano plot of 525 down‐regulated and 880 up‐regulated DEGs between the DOX+siNC and DOX+siAD19 groups. D) KEGG enriched biological process and signaling pathways based on DEGs between the two groups. E‐H) GSEA of the selected terms in DOX+siAD19 group compared to DOX+siNC control. I) Potential schematic signaling pathways affected by ADAM19 knockdown based on RNA sequencing data. J, K) The phosphorylation protein level of PI3K/AKT in mouse chondrocytes J) and relative grey level of phospho‐PI3K/AKT protein bands normalized to total PI3K/AKT K). Data are presented as means ± SD of at least 3 independent experiments. ***p < 0.001 versus untreated control, ^##^ *p* < 0.01, ^###^ *p* < 0.001 versus DOX+siNC group.

To further illuminate the potential molecular mechanism of ADAM19 on chondrocyte senescence, RNA sequencing was performed. Principal component analysis (PCA) showed the gene expression patterns of si‐ADAM19 treated senescent chondrocyte clustered away from NC siRNA (**Figure** **4**A). Unbiased hierarchical clustering heatmap and volcano plot revealed si‐ADAM19 treatment not only reduced cell cycle‐and SASP‐related gene expression but also promote cell proliferation and matrix synthesis (Figure 4B,C). To explore the pathway changes in cellular senescence after si‐ADAM19 treatment, Kyoto Encyclopedia of Genes and Genomes (KEGG) analyses were performed. ADAM19 knockdown significantly down‐regulated multiple inflammation‐related pathways such as NOD‐like and Toll‐like receptor signaling pathways, and induced cell cycle and DNA repair processes (Figure 4D). We next assessed the senolytic effect of ADAM19 knockdown on senescent chondrocytes by examining SenMayo gene set, which was recently reported to identify SnCs and predicted senescence‐associated pathways.^[^ ^23^ ^]^ Gene Set Enrichment Analysis (GSEA) showed SenMayo was significantly reduced in senescent chondrocyte following si‐ADAM19 treatment (Figure 4E). In addition, ADAM19 knockdown significantly enriched down‐regulated genes in inflammatory response (Figure 4F) and positively mediated DNA replication/repair‐and extracellular matrix synthesis‐associated pathways in senescent chondrocytes (Figure 4G,H). The PI3K/AKT/mTOR signaling pathway plays a critical role in the regulation of cellular senescence. Intervention of the key components in the pathway could effectively attenuate senescence‐associated diseases and extend lifespan.^[^ ^24^ ^]^ Under the context of OA, inhibition of PI3K/AKT/mTOR pathway promotes autophagy in chondrocyte and reduces the inflammatory response in the damaged joint.^[^ ^25^ ^]^ Besides, Chen, et al.^[^ ^15^ ^]^ recently identified an oncogenic lncRNA (LINC00511) that can promote tumor progression by modulating ADAM19 and the downstream PI3K/AKT pathway. Here, we tried to determine whether PI3K/AKT pathways were involved in ADAM19‐mediated cellular senescence. Analyzing the up and downstream relationships of the genes in the reference pathway derived from the KEGG database suggested that ADAM19 may exert senolytic effect by inhibiting the PI3K/AKT signaling pathway and down‐regulate the expression of pro‐inflammatory SASP by targeting activator protein 1 (AP1), which was considered as a regulatory transcription factor of cellular senescence^[^ ^26^ ^]^ (Figure 4I). Western blot analysis further revealed ADAM19 knockdown effectively attenuated phosphorylation of PI3K/AKT in senescent chondrocytes (Figure 4J,K). These results suggested PI3K/AKT signaling pathway might be a possible underlying mechanism by which ADAM19 knockdown mediated the rejuvenation of senescent chondrocytes.

### 2.5 Characterization and Function of Senescence‐Targeting Chondrocyte Rejuvenating Nanoparticle with si‐ADAM19 Delivery

**Figure 5:**
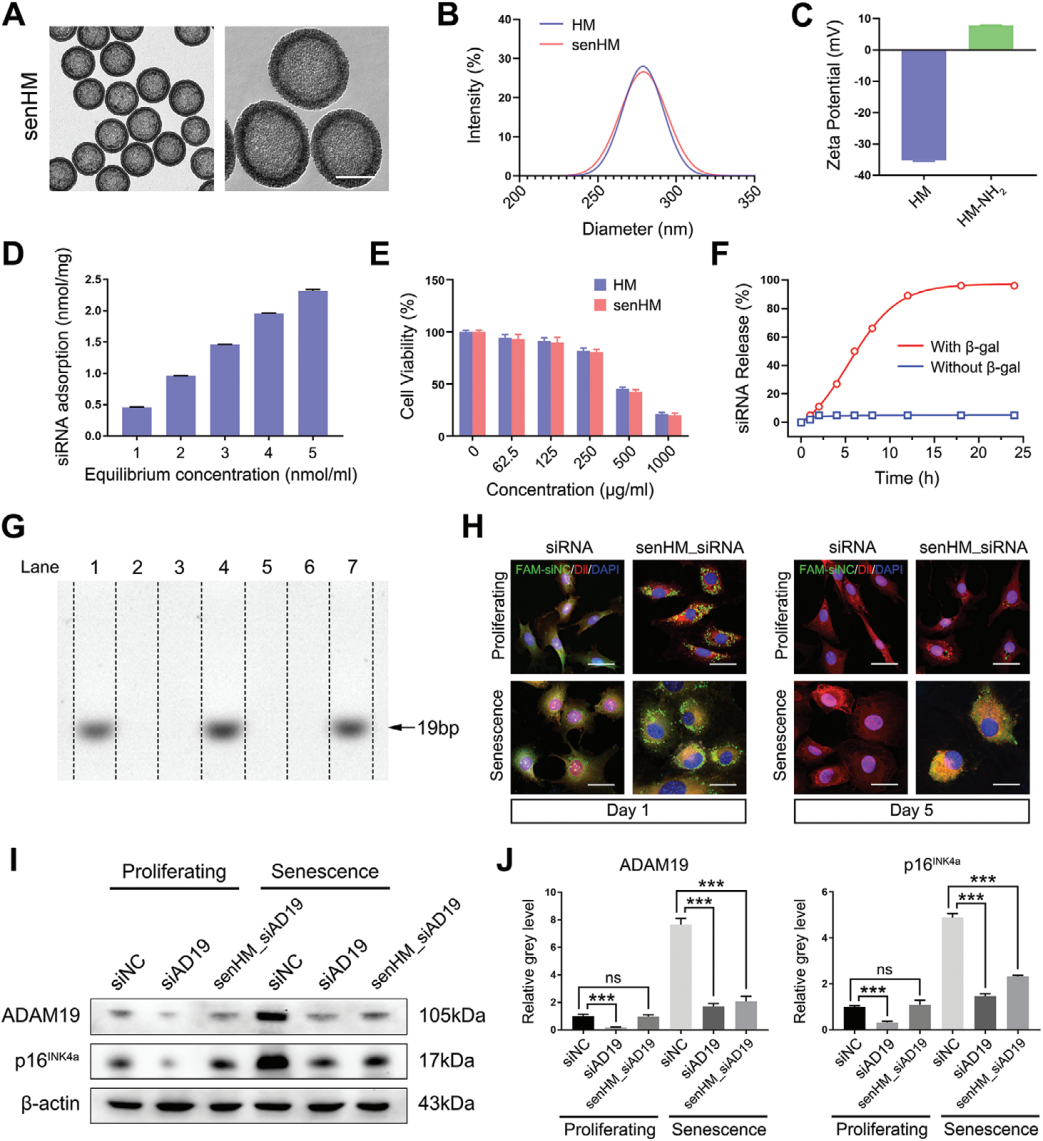
Characterization and function of senHMSNs. A) TEM images of senHMSNs (senHM). Scale bar, 200 nm. B) Hydrodynamic diameter distribution of HMSNs (HM) or senHM. C) Zeta potential of HM or amino‐functionalized HM (HM‐NH_2_) suspended in RNase‐free water (pH = 7.0). D) The siRNA adsorption of senHM under various siRNA equilibrium concentrations. E) Cell viabilities of the mouse chondrocytes incubated with HM and senHM for 48 h. F) Release profiles of siRNA from siRNA‐loaded senHM in the absence and presence of β‐gal in PBS (pH = 7.5). G) Agarose gel electrophoretic analysis of siRNA protective effect by senHM. Lane1: naked siRNA; Lane2: naked siRNA treated with RNase A; Lane3: senHM_siRNA; Lane4: senHM_siRNA treated with β‐gal; Lane5: senHM_siRNA first treated with β‐gal and then with RNase A; Lane6: senHM_siRNA treated with RNase A; Lane7: senHM_siRNA first treated with RNase A, then separated the particles and digested by β‐gal. H) Representative confocal images of naked FAM‐labeled siRNA or siRNA‐loaded senHM (senHM_siRNA) incubated with proliferating or senescent mouse chondrocytes. Scale bar, 50 µm. I, J) The protein level of ADAM19 in proliferating or senescent mouse chondrocytes treated with siNC, siAD19, and siAD19‐loaded senHM (senHM_siAD19) (I) and relative grey level of ADAM19 and p16^INK4a^ protein bands normalized to β‐actin (J). The quantity of senHM_siRNA for in vitro application is 21.7 µg mL^−1^, with equivalent siRNA concentration of 50 nM. Data are presented as means ± SD of at least 3 independent experiments. ***p < 0.001, no significance (ns).

To deliver sufficient and intact therapeutic siRNA into senescent chondrocytes, we developed a senescence‐targeting siRNA delivery system with high loading capacity. Mesoporous silica nanoparticles (MSNs) have unique pore structure that enables greater cargo loading capacity than other nanomaterials and provide a stable nucleic acid protection environment, making them particularly suitable for siRNA delivery.^[^ ^27^ ^]^ In this work, hollow MSNs (HMSNs) were used as siRNA carrier, which provide ultra large capacity for cargo loading.^[^ ^28^ ^]^ Galacto‐oligosaccharide (GOS) was coated on the surface of HMSNs (senHMSNs) to achieve senescence‐responsive cargo release.^[^ ^29^ ^]^ Multiple techniques were used to characterize the material: The TEM images showed the morphology of senHMSNs presented a hollow structural characteristic (**Figure** **5**A) and DLS confirmed the average diameter of senHMSNs was ≈280 nm (Figure 5B). The pore size of the HMSNs was ≈4.3 nm and the surface area was characterized as 679.6 m^2^ g^−1^ (Figure S3A, B, Supporting Information). For GOS encapsulation, we functionalized the surface of HMSNs with amino group (─NH_2_). The feature absorption peak of the amino group (─NH_2_) modified on the HMSNs detected by Fourier Transform Infrared (FT‐IR) spectra was observed near 1560 cm^−1^, which disappeared after GOS encapsulation (Figure S3C, Supporting Information). The zeta potential of the HMSNs was also reversed from a negatively charged (−35.17 ± 0.25 mV) to a positively charged (7.87 ± 0.15 mV) after amino‐functionalization, which offering electrostatic force for siRNA loading (Figure 5C). The amount of therapeutic siRNA adsorbed in senHMSNs reached 2.3 nmol mg^−1^ HMSN when the equilibrium concentration of siRNA was 5 nmol mL^−1^ (Figure 5D). In comparison, the siRNA loading amount of MSNs with ultra‐large pores was 1.25 pmol siRNA/ug MSN.^[^ ^30^ ^]^ Before further biomedical applications, the viability of chondrocytes treated with different concentrations of HMSNs was determined. senHMSNs exhibited extremely low cytotoxicity toward mouse chondrocytes at concentrations no more than 250 µg mL^−1^ (Figure 5E). The senescence‐responsive siRNA release property of the senHMSNs was also measured. In the presence of β‐gal, the release of siRNA loaded in senHMSNs increased in a time‐dependent manner and was almost released completely after 12 h, while the siRNA remained in the senHMSNs in the absence of β‐gal (Figure 5F). In addition, the senHMSNs could effectively shield siRNA payload from RNase degradation (Figure 5G). To further evaluate whether the senHMSNs could target delivery of siRNA into SnCs, Dil stained proliferating or senescent chondrocytes were incubated with FAM‐labeled siRNA‐loaded senHMSNs to monitor the delivery. Interestingly, siRNA‐loaded senHMSNs could be well uptake by chondrocytes and continuously release siRNA in the SnCs for at least 5 days (Figure 5H). Moreover, si‐ADAM19‐loaded senHMSNs (senHMSN_si‐ADAM19) could selectively knockdown ADAM19 and inhibit p16^INK4A^ protein expression with high efficiency in senescent rather than proliferating chondrocytes (Figure 5I,J). All these demonstrated that the senHMSN_si‐ADAM19 could efficiently knockdown target gene in SnCs and attenuated chondrocyte senescence.

### 2.6 senHMSN_si‐ADAM19 Promotes Rejuvenating Hyaline Cartilage Regeneration in Mouse Post‐Traumatic OA

**Figure 6:**
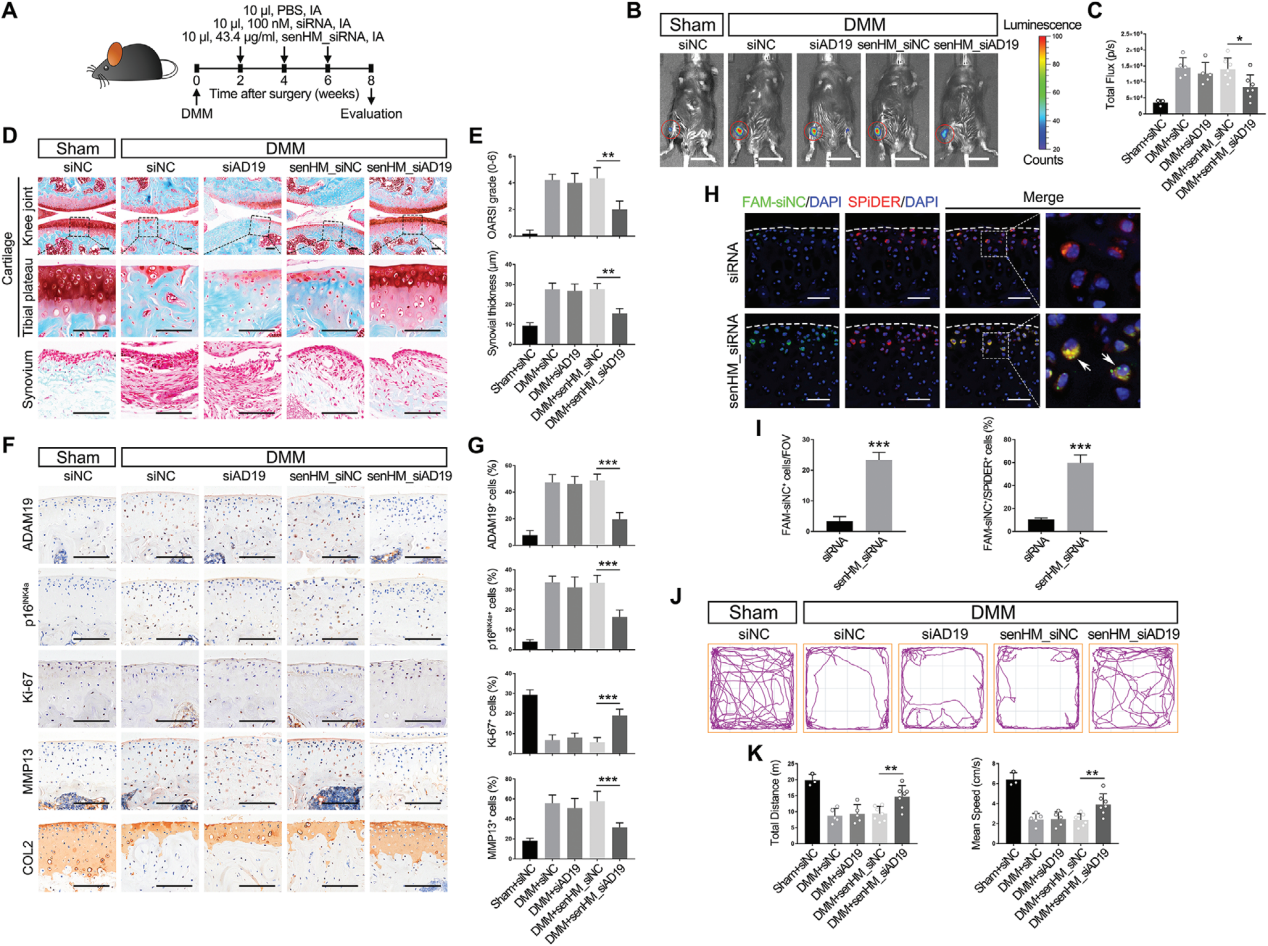
Therapeutic effects of senHMSN_si‐ADAM19 in post‐traumatic OA mice. A) Schematic diagram of IA injection in p16‐3MR mice after DMM surgery. B, C) Representative bioluminescence images of Sham or DMM p16‐3MR mice after IA injection (B) and quantification of the luminescence signaling (total flux) in knee joint (C). Scale bars, 2 cm. *p < 0.05. D, E) Representative images of Safranin O/Fast Green staining for histological analysis (D) and quantification of cartilage destruction in medial tibial plateau and anterior femoral synovial membrane thickness (E). F, G) Representative immunohistochemistry images of ADAM19, p16^INK4a^, MMP13, Ki‐67, and COL2 in articular cartilage (F) and quantification of ADAM19‐, p16^INK4a^‐, Ki‐67‐, and MMP13‐positive cells (G). Scale bar, 100 µm. H, I) Representative confocal images of IA injection of naked FAM‐labeled siRNA or senHM_siRNA in mice after DMM surgery (H) and quantification of FAM‐positive cells (I). FOV, field of view. Scale bar, 50 µm. Dotted lines indicate the cartilage surface. J, K) Representative images of open field test (J) and total distance and average movement speed of mice (K). Sham+siNC, *n* = 3; DMM+siNC and DMM+siAD19, *n* = 5; DMM+senHM_siNC and DMM+senHM_siAD19, *n* = 7. Data are presented as means ± SD. *p < 0.05, **p < 0.01, ***p < 0.001.

We next wondered whether senHMSN_si‐ADAM19 could ameliorate OA in mice. We performed DMM surgery to mimic post‐traumatic OA in p16‐3MR transgenic mice, in which allowed non‐invasively detecting of SnCs.^[^ ^31^ ^]^ Beginning 2 weeks after surgery, p16‐3MR mice were intra‐articular (IA) injected with naked siRNA or siRNA‐loaded senHMSNs once every two weeks (**Figure** **6**A). Surprisingly, senHMSN_si‐ADAM19 treatment significantly lowered the p16‐driven luminescence signal in the joint region of DMM mice, indicating a significant reduction of SnCs accumulation (Figure 6B,C). Histological assessment also showed surgical‐induced cartilage destruction and synovial inflammation were ameliorated after senHMSN_si‐ADAM19 treatment (Figure 6D,E). At molecular level, senHMSN_si‐ADAM19 efficiently knocked down ADAM19, reduced the number of p16^INK4a^‐positive SnCs and MMP13‐expressing cells, and further restored the impaired proliferation phenotype of chondrocytes in DMM mice (Figure 6F,G). These results implied the senescent phenotype of osteoarthritic cartilage was effectively attenuated. Moreover, senHMSN_si‐ADAM19 treatment also significantly increased the content of cartilage matrix such as collagen type II in the damaged joint (Figure 6D,F). To further examine the senescence‐responsive siRNA release of the senHMSNs in vivo, senHMSN_si‐ADAM19 was injected into the DMM‐operated joints. SnCs were signed by SPiDER fluorescent probe, which can label SA‐β‐gal‐positive cells.^[^ ^32^ ^]^ Noteworthy, the cells co‐stained with FAM‐labeled siRNA and SPiDER probe were only detected in the joints injected with senHMSNs, indicating siRNA was specifically released in senescent chondrocytes (Figure 6H,I). Joint function assessed by ANY‐maze monitoring revealed that senHMSN_si‐ADAM19 improved the total distance or mean speed loss caused by the DMM surgery (Figure 6J,K). Furthermore, senHMSN_si‐ADAM19 treated mice showed no pathological abnormalities in the major organs and no differences in body weight throughout the administration period, indicating high biocompatibility of the agents for in vivo OA therapy (Figure S4, Supporting Information). Taken together, senHMSN_si‐ADAM19 could effectively promote the regeneration of hyaline cartilage and alleviate the progression of experimental OA in mice.

## 3 Discussion

Experimental and clinical evidence has established that cellular senescence and the accumulation of SnCs are involved in the progression of various age‐related chronic diseases.^[^ ^24^, ^33^ ^]^ Thus, therapies targeting SnCs can improve organ dysfunction in age‐associated disorders. However, unlike other tissues, cartilage is a special tissue with an abundant matrix but poor replicative ability of chondrocytes, leading to a fragile regenerative capacity.^[^ ^34^ ^]^ The characteristic of cartilage implies that conventional senolytic therapies may accelerate chondrocyte depletion and easily disrupt the balance of cartilage regeneration in the context of OA, while the SASP inhibition strategy also unable to kill SnCs and form proliferative chondrocytes.

To investigate the genetic regulators of cellular senescence in SnCs, we performed genome‐wide CRISPR screening in senescent human mesenchymal stem cells, in which ADAM19 deficiency significantly alleviated the SASP of SnCs. As a kind of “Sheddases”, ADAM19 is known to proteolytic cleavage of the membrane‐bound extracellular domain. Dysregulation of transmembrane ectodomain shedding may contribute to the tumor progression, inflammatory‐, and metabolic‐associated diseases as previously mentioned.^[^ ^15^, ^16^, ^17^, ^35^ ^]^ Recently, Kong, et al.^[^ ^36a,b^ ^]^ have shown synovial mesenchymal stem cell derived exosomal microRNA‐320c enhanced cell proliferation and chondrogenesis by reducing ADAM19 expression. In this work, we reinforced ADAM19 for the first time as a cartilage rejuvenator in OA pathology. Our results showed the up‐regulation of ADAM19 under the context of cellular senescence may impair chondrocyte proliferation and matrix synthesis. Existing gene therapies targeting chondrocyte senescence mainly focus on the matrix degradative enzymes,^[^ ^37^ ^]^ which alleviated the progression of OA through reducing cartilage degradation. By contrast, gene therapy targeting ADAM19 may serve as an attractive strategy to ameliorate cellular senescence. Intriguingly, knockdown ADAM19 in senescent chondrocyte not only attenuated the chondrocyte senescence, but also promoted the regeneration phenotype of chondrocyte including cell proliferation and extracellular matrix synthesis. In other words, the therapy targeting ADAM19 generated chondrocytes with a rejuvenating regenerative phenotype, which could proliferate and synthesize cartilage matrix. In conclusion, our senotherapy may effectively promote the regeneration of osteoarthritic cartilage and prevent the progression of OA to the terminal stage.

Delivering therapeutic siRNA in SnCs within articular cartilage remains a significant challenge.^[^ ^38^ ^]^ MSN‐based senescence‐targeting delivery strategy has been shown to prevent cargo delivery within non‐SnCs and helps to reduce the drug‐related toxicity in normal cells.^[^ ^29^ ^]^ However, the delivery system narrated above has been reported comprising fluorophores or chemotherapeutic drugs, whether it can be loaded with therapeutic siRNA and applied to the OA disease scenario remains to be further investigated. The senHMSNs we developed could be well uptake by chondrocytes and continuously release siRNA only in SnCs but not in proliferating cells. Notably, senHMSN_si‐ADAM19 showed up to ≈75% knockdown of ADAM19 expression in senescent chondrocytes, which was comparable to the commercially available siRNA transfection reagents, and down‐regulating the expression of p16^INK4a^. Given that senHMSN_si‐ADAM19 could specifically knockdown the expression of target genes in senescent chondrocyte and ameliorate cellular senescence in vitro, we further tested the efficacy of senHMSN_si‐ADAM19 in post‐traumatic OA mice. Surprisingly, IA injection of senHMSN_si‐ADAM19 not only attenuated p16‐driven luminescence, reduced the number of p16^INK4a^‐and MMP13‐positive cells, but also regenerated hyaline cartilage in the osteoarthritic joints. In addition, we also visualized senescent chondrocytes within the cartilage as a preferential target for senHMSNs delivery of siRNA in vivo. The development of OA is not only related with chondrocyte senescence but also affected by mechanical friction between the osteoarthritic cartilages.^[^ ^39^ ^]^ Apart from targeted drug delivery, functional MSNs can also be designed to mimic the hydration lubrication with articular cartilage.^[^ ^40^ ^]^ Despite the senHMSNs was not specifically optimized for joint lubrication, the high siRNA loading capacity of the delivery system allowed for low doses of IA administration, thereby avoiding potential adverse effects on joint friction. Our results first highlighted the advantages of the senHMSNs in delivering therapeutic siRNA in SnCs and its applicability in OA disease scenario.

Although we confirmed the senolytic effect and regenerative potential of the senHMSN_si‐ADAM19 agents on hyaline cartilage in a mouse post‐traumatic OA model, unable to further test the efficacy of the agents in large animals with anatomy and mechanics similar to the human joints might be a potential weakness. Besides, considering some non‐SnCs, such as macrophages, may exhibit abnormally increased SA‐β‐gal activity,^[^ ^41^ ^]^ we further explore how the delivery system interacts with macrophages. The activity of SA‐β‐gal and the expression of ADAM19 were elevated in both classically activated (M1) and alternatively activated (M2) macrophages. However, no significant knockdown of target gene was observed in macrophages after treatment with naked si‐ADAM19 or senHMSN_si‐ADAM19 (Figure S5, Supporting Information), which may be due to the obstacles in gene editing and engineering in primary macrophages.^[^ ^42^ ^]^ Our results showed the GOS encapsulation senescence‐targeting strategy may not be suitable for nucleic acid drug delivery in macrophages.

## 4 Conclusion

To our knowledge, this is the first study to elucidate the critical role of ADAM19‐targeting therapy in rejuvenating senescent chondrocyte and osteoarthritic cartilage regeneration. Knockdown of ADAM19 resulted in the attenuation of cellular senescence and the formation of proliferative extracellular matrix‐synthesizing chondrocytes. In addition, we developed a senHMSN_si‐ADAM19 agent, which could target deliver therapeutic siRNA into SnCs and rejuvenate hyaline cartilage in experimental OA mice (Figure S6, Supporting Information). ADAM19‐based gene therapy combining with senescence‐targeting siRNA delivery may provide a novel approach for in vivo drug delivery to mitigate OA or other age‐related diseases in the future.

## 5 Experimental Section

### Experimental OA Mouse Model

C57BL/6 mice were purchased from Shanghai Laboratory Animal Company (SLAC). p16‐3MR mice were generously donated by Judith Campisi, Buck Institute for Research on Aging, USA. Animal experiments were conducted in accordance with standard ethical guidelines and approved by the animal ethics committee of Zhejiang university (ZJU20210132). Destabilization of the Medial Meniscus (DMM) and sham surgery was performed on right hind limbs of 10‐week‐old male mice as previously described.^[^ ^43^ ^]^ Briefly, animals were given general anesthesia with 3% isoflurane, and hind limbs were shaved to expose the surgical field. A 5 mm longitudinal incision was made along the distal patellar tendon with surgical scissors. Identified the meniscotibial ligament of the medial meniscus and severed this ligament carefully with a microscalpel under stereomicroscope. For sham surgery, identified the ligament but did not sever. The incisions were closed after the tissue debris were flushed with sterile saline. All surgical procedures were followed the aseptic principle. 8 or 12 weeks after surgery, the mice were euthanized and the joints were collected for further analysis.

### Human Specimens

Human tissues were collected according to protocols approved by the ethics committee of Zhejiang Provincial People's Hospital (IRB NO. 2019KY072). All subjects have signed a written informed consent approved by the ethics committee before the surgery. Patients who underwent total knee arthroplasty (TKA) with symptomatic OA were selected for this study. Additionally, patients with previous instrumented knee surgery or other joint disorders, such as active infection, bone metabolic diseases, or tumors were excluded. Patients with no history of knee OA but required amputation were considered as normal control. Samples used for primary cell extraction were collected from OA patients with no history of pharmacologic treatment within 3 years.

### Primary Cells Extraction and Chemotherapy‐Induced Cell Senescence Model

Mouse primary chondrocytes were isolated from the femoral condyles and tibial plateaus of 5‐day‐postnatal C57BL/6 mice. The methods of mouse primary chondrocytes extraction were as previously described.^[^ ^44^ ^]^ Human primary chondrocytes were isolated from the relatively undamaged areas of the lateral tibial plateau of human cartilage. The human primary chondrocytes were extracted according to the method as Goldring^[^ ^45^ ^]^ described. Primary chondrocytes were maintained in DMEM/F‐12 with 10% FBS and 1% antibiotics and used for the experiments at 80% confluence. The cells were incubated in a humidified atmosphere of 5% CO_2_ and 3% O_2_ at 37 °C. For chemotherapy‐induced cell senescence model, chondrocytes at 50% confluence were treated with different concentrations of doxorubicin (DOX) for 48 h, and cells were continued to be cultured with fresh DOX‐free medium for another 7 days to assess cell senescence. For ionizing radiation (IR)‐induced cellular senescence model, chondrocytes at 50% confluence were irradiated with a single dose of 10 Gy (X‐ray) at a dose rate of 2 Gy min^−1^ using RS2000 irradiator (RAD SOURCE, USA). After IR exposure, cells were continued to be cultured for another 7 days. Culture medium was refreshed every 2 days.

### Small Interfering RNA (siRNA) Transfection

Chondrocytes were seeded at 50% confluence in 12 well plate (Day 0) and treated with 200 nM DOX for 48 h (Day 2–4) to induce senescence. ADAM19 siRNA or negative control (NC) siRNA (RIBOBIO, China) at a final concentration of 50 nM combined with 10 mg mL^−1^ Lipofectamine RNAiMAX (Invitrogen, USA) were transfected every two days (Day 5, 8, 11). Samples were collected (Day 12) for subsequent experiments.

### Quantitative Real‐Time PCR (qPCR) Analysis

The total RNA was extracted using RNAiso Plus reagent (Takara, Japan) and then reverse‐transcribed using PrimeScript RT Master Mix (Takara). qPCR was then performed on Light Cycler 480II apparatus (Roche, Germany) using the SYBR Green qPCR Master Mix (Takara) as follows: 95 °C for 10 min (activation), followed by 40 cycles of 95 °C for 10 s, 60 °C for 30 s, and 72 °C for 30 s (amplification), and a final extension at 72 °C for 90 s. Specificity of amplification was confirmed by melting curves. Relative expression of target genes was normalized to β‐actin and calculated using the 2^‐ΔΔCt method. Primer sequences used in experiments were listed in Table S1 (Supporting Information).

### RNA Sequencing

RNA for sequencing was isolated from mouse primary chondrocytes and 50 bp single‐ended sequencing were performed by BGI Genomics Co., Ltd (Shenzhen, China). Low‐quality reads were filtered using Trim Galore(v0.6.4) and mapped to mouse genome (m38. p6) using HISAT2(v2.1.0) with default parameters. Gene expression was calculated in counts per million (CPM) using featureCounts (v1.6.1). DESeq2 was used to identify differentially expressed genes (log fold change of 1, adjusted P value of 0.05). Heatmaps were created using the heatmap R package (v1.0.12) from manually selected genes. KEGG enrichment were generated with Bioconductor's cluster Profiler R package (v3.16.1) and their distribution was plotted using ggplot 2 R package (v3.3.2).

### Western Blot Analysis

Total protein from the cultured cells was extracted using RIPA lysis buffer (Beyotime, China) with protease‐inhibitor cocktail. The protein concentration was quantified using a BCA protein assay kit (Beyotime). Protein samples were separated by 10% SDS‐PAGE gels and electrotransferred onto PVDF membranes (Millipore, USA). The membrane was blocked in 5% (w/v) non‐fat dry milk in TBST buffer for 1 h at room temperature and then incubated with primary antibody at 4 °C overnight. Then, the membrane was incubated with HRP‐linked secondary antibody for 1 h. Protein bands were visualized using ChemiDoc imaging system (BIO RAD, USA) and quantified using ImageJ software (National Institution of Health, USA).

### Immunofluorescence

Immunofluorescence staining was performed on 10 µm thick frozen sections. Sections were first stained with primary antibodies: anti‐ADAM19 (ab191457, Abcam), p16^INK4a^ (ab241543, Abcam), and Ki67 (ab16667, Abcam) and then fluorescent secondary antibodies. After nuclear counterstained with DAPI for 15 min, cells were visualized using a laser scanning confocal microscope (OLYMPUS, Japan). All staining procedure was performed in the absence of visible light to prevent photosensitivity.

### Senescence‐Associated β‐Galactosidase (SA‐β‐Gal) Staining

Staining was performed using SA‐β‐gal staining kit (Beyotime) according to the manufacturer's protocol. Blue‐stained cells under light microscope were identified as SA‐β‐gal‐positive senescent cells (SnCs).

### Alcian Blue Staining and Glycosaminoglycan (GAG) Content Quantification

Chondrocytes were seeded at 80% confluence in 12 well plate and treated with DOX to induce senescence. Cells were then transfected with ADAM19 or NC siRNA three times after DOX treatment and were continued to be cultured with fresh medium for another 14 days for subsequent analysis. Alcian blue staining: cells were fixed with 4% paraformaldehyde (PFA) for 15 min at room temperature and then stained with Alcian blue solution (Beyotime, China) for 30 min; GAG quantification: cells were lyophilized and digested in 200 µL papain buffer at 65 °C overnight. 20 µL supernatant was then mixed with 100 µL of 1,9‐dimethylmethylene blue (DMMB) solution. The absorbance at 525 nm was detected by microplate reader and compared with chondroitin sulfate sodium standard (Sigma Aldrich) to quantify the GAG content per well.

### Preparation of Senescence‐Targeting siRNA Delivery System

Synthesis of hollow mesoporous silica nanoparticles (HMSNs): ethanol (74 mL), ammonia (3.14 mL), deionized water (90 mL), and tetraethoxysilane (TEOS, 6 mL) were mixed and stirred for 1 h to prepare the silica core. The products collected by centrifugation were washed with ethanol and deionized water. Cetyltrimethylammonium Chloride (CTAC, 2.00 g) and triethanolamine (TEA, 0.4 g) was dissolved in 90 mL deionized water and then added the silica core. After that, TEOS (1.8 mL) was added dropwise to the solution and stirred at 80 °C for 2.5 h. The products collected by centrifugation were dispersed into 60 mL sodium carbonate solution and stirred for 4 h to corrode the silica core. After washed with deionized water for several times, the products were dried at 60 °C for 24 h and calcined at 550 °C for 5 h to prepare the final products. Preparation of amino‐functionalized HMSNs (HMSN‐NH_2_): HMSNs (0.2 g), toluene (15 mL) and aminopropyltriethoxysilane (APTES, 0.8 mL) were mixed in a flask purged with N_2_ and stirred at 85 °C for 24 h. The products collected by centrifugation were washed with toluene and ethanol. Before siRNA packaging, the HMSN‐NH_2_ was disinfected in 75% ethanol for 1 h and then washed 5 times with RNase‐free water to remove residual ethanol in Vertical Flow Clean Bench. siRNA packaging within the HMSN‐NH_2_: 20 µL siRNA solution and 2 mg HMSN‐NH_2_ were mixed into a 1.5 mL EP tube, then 1 mL RNase‐free water was added. After thoroughly mixed by vortex, the mixture was continuously stirred at 270 rpm for 2 h. Galacto‐oligosaccharide (GOS) encapsulation: the siRNA‐loaded HMSN‐NH_2_ were suspended in 20 mg mL^−1^ commercially available GOS solution (dissolved in RNase‐free water) and stirred at 4 °C overnight. siRNA packaging and GOS encapsulation were performed according to the aseptic operation procedure. The final products (senHMSNs) were washed with deionized water for several times and stored in 4 °C for further use.

### Material Characterization

The morphological analysis of the senHMSNs was performed on Tecnai G2 spirit bio‐twin transmission electron microscope (TEM) (FEI, USA). Surface characterization of HMSNs was assessed by Automatic Fast Specific Surface and Porosity Analyzer (Micromeritics, USA). The hydrodynamic size distribution and surface charge (zeta potential) was determined by Dynamic Lighter Scattering (DLS) and Electrophoretic Light Scattering (ELS) using the Zetasizer Nano ZS particle sizer (Malvern, England).

### Loading Capacity of siRNA in HMSNs

2 mg HMSN‐NH_2_ was added to 1 mL siRNA solution with different equilibrium concentrations, the concentration of siRNA in binding solution before and after HMSNs adsorption was measured by NANODROP ONE spectrophotometer (Thermo Scientific, USA) and siRNA adsorption was calculated according to the differences of siRNA concentration. The adjusted concentration of siRNA was calibrated by the standard curve.

### Beta‐Galactosidase (β‐Gal) Responsive siRNA Release

β‐gal responsive siRNA release profile of senHMSNs: 10 mg siRNA‐loaded senHMSNs (senHMSN_siRNA) was suspended in 4 mL RNase‐free water and then added 1 mL β‐gal solution (50 mg mL^−1^) at pH = 7.5. siRNA released in the solution were determined using NANODROP ONE spectrophotometer at 0, 1, 2, 4, 6, 8, 12, 18, and 24 h after β‐gal treatment at room temperature.

### siRNA Protection Assessment

Agarose gel electrophoresis was used to detect the protective effect of senHMSNs on siRNA. Samples of RNase A or β‐gal treated naked siRNA or senHMSN_siRNA were separated on 1% agarose gels containing 0.01% Gelred for 30 min at 100 V. The dose of RNase was about 0.25% (w/w) with incubation at 37 °C for 30 min. The dose of β‐gal was ≈25 mg mL^−1^ with incubation at 37 °C for 1 h.

### Cellular Uptake Study

1 × 10^5^ proliferating or DOX‐treated senescent chondrocytes were seeded in 35 mm glass bottom dishes (Greiner Bio‐One, Germany) and the cells were incubated with senHMSN_siRNA for 24 h. Then the cells were fixed with 4% PFA and counterstained nuclear with DAPI, the engulfment of senHMSN_siRNA by chondrocytes and release of siRNA was visualized using a laser scanning confocal microscope (OLYMPUS, Japan).

### Intra‐Articular (IA) Injection

10 µL of naked ADAM19 or NC siRNA and ADAM19 or NC siRNA‐loaded senHMSNs agents were intra‐articular (IA) injected carefully using a 33G 25 µL microsyringe (Hamilton, USA) once every two weeks starting from 2 weeks after DMM surgery. The sham mice injected with naked NC siRNA were considered as the normal control. The quantity of senHMSN_siRNA for in vivo application was converted according to the loading capacity of siRNA in HMSNs, which equivalent to naked siRNA concentration of 100 nM. The mice were sacrificed 8 weeks after DMM surgery and the knee joints were collected for further analysis.

### Bioluminescence Imaging

For in vivo luminescence imaging, p16‐3MR mice were first anesthetized with isoflurane. Mice bilateral knee joints were both IA injected with 10 µL (150 µg mL^−1^) of RediJect Coelenterazine h (PerkinElmer, 760 506). After incubating in thermotank for 10 min, the luminescence of knee joints was quantified by IVIS Spectrum image system (PerkinElmer, USA) using the following parameters: Field of View: D, Medium Binning, Exposure Time: 2 min.

### Histological Evaluation and Immunostaining

Specimens were fixed in 4% PFA, decalcified in 12% EDTA (pH 7.0) and embedded in paraffin. Subsequently, the embedded samples were sectioned into 5 µm sections, which were randomly selected for Safranin O/Fast Green staining. Osteoarthritis Research Society International (OARSI) scoring system^[^ ^46^ ^]^ was graded blind by two experienced experimenters. For immunohistochemistry, the sections underwent antigen retrieval and were subsequently blocked for endogenous peroxidase activity. Nonspecific antibody binding was blocked using appropriate serum mixtures. After that, sections were incubated with the following primary antibodies: anti‐ADAM19 (ab191457, Abcam), p16^INK4a^ (ab241543, Abcam), MMP13 (ab219620, Abcam), and COL2 (sc‐52658, Santa Cruz) at 4 °C overnight and then stained with HRP‐linked secondary antibodies at room temperature for 1 h. SPiDER fluorescent probe labeling of SA‐β‐gal in SnCs was performed using Cellular senescence detection kit (DOJINDO, JAPAN) according to the manufacturer's protocols. Positive staining cells were counted in the articular cartilage of each sample and the percentage of positive cells was calculated using ImageJ software.

### Open Field Test

Open field test was analyzed by a video tracking system (ViewPoint, Shanghai, China). The mice were adapted to the experimental environment for at least 1 h before the experiment start. The environment was kept quiet during the experiment. Each mouse was placed on the outer zone of the chamber to avoid stressful situation exposure and allowed to explore freely for 5 min. The mobility parameters of mice were recorded automatically and analyzed using ANY‐maze software (Stoelting, USA). Experiments and data collection were performed at the same time in each day and the participated by the same experimentalists.

### Statistics

Data were represented as averages ±SD for at least three independent experiments. Statistical significance (P values) was determined using either Student t test with two‐tailed distributions or one‐way ANOVA with Tukey's multiple‐comparisons test unless otherwise stated. Statistical analysis was performed in R (v3.6.3) and *p* < 0.05 was considered statistically significant.

## Conflict of Interest

The authors declare no conflict of interest.

## Supporting information



Supporting Information

## Data Availability

Research data are not shared.
