# Supplementary material for: Rejuvenating Hyaline Cartilage with Senescence‐Targeting Si‐ADAM19 Delivery for Osteoarthritis Therapy
Source: Adv Sci (Weinh). 2025 Feb 10;12(13):2414419. doi: 10.1002/advs.202414419 (PMC11967805; doi:10.1002/advs.202414419)
Supplement: Supplementary file 1 — Supporting Information [file ADVS-12-2414419-s001.docx]

Supporting Information

**Rejuvenating Hyaline Cartilage with Senescence-Targeting Si-ADAM19 Delivery for Osteoarthritis Therapy**

Jiasheng Wang^1,2*^, Peng Guo^1,2*^, Dongmei Wu^1,2^, Junzhi Yi^1,2^, Qi Jiang^1,2^, Jiajie Hu^1,2,4#^, and Hongwei Ouyang^1,2,3,4#^

1. Department of Sports Medicine of the Second Affiliated Hospital, and Liangzhu Laboratory, Zhejiang University School of Medicine, Hangzhou, 310058, China.

2. Dr. Li Dak Sum & Yip Yio Chin Center for Stem Cells and Regenerative Medicine, Zhejiang University School of Medicine, Hangzhou, 310058, China.

3. Zhejiang University-University of Edinburgh Institute, Zhejiang University School of Medicine, Haining, 310058, China.

4. China Orthopedic Regenerative Medicine Group (CORMed), Hangzhou, China.

**^#^ Corresponding Author:** Hongwei Ouyang, Ph.D

**Address:** 866 Yuhangtang Road, Hangzhou 310058, China.

**E-mail:** hwoy@zju.edu.cn

**^#^ co-Corresponding Author:** Jiajie Hu, Ph.D

**Address:** 866 Yuhangtang Road, Hangzhou 310058, China.

**E-mail:** jiajie_hu@zju.edu.cn

* Jiasheng Wang and Peng Guo contributed equally to this study.

**Key words**

osteoarthritis(OA); rejuvenating; cartilage regeneration; a disintegrin and metalloproteinase 19 (ADAM19); cellular senescence; siRNA Delivery

**Table**

Table S1. Primer sequences of qRT-PCR

| Species | Genes |  | Primer sequence (5′–3′) |
| --- | --- | --- | --- |
| human | ADAM19 | Forward primer  Reverse primer | CTCTGCTTGCTGGCGTTTG  TCACGGGGCTTTCTGAAGTC |
|  | CDKN2A | Forward primer  Reverse primer | GGGTCGGGTAGAGGAGGTG  GCTGCCCATCATCATGACCT |
|  | CDKN1A | Forward primer  Reverse primer | AGTCAGTTCCTTGTGGAGCC  CATTAGCGCATCACAGTCGC |
|  | LMNB1 | Forward primer  Reverse primer | TCCGTTCCTCTAAACGCCAG  GGACCGTGATAAGGAGGGGA |
|  | MMP13 | Forward primer  Reverse primer | GCACTTCCCACAGTGCCTAT  AGTTCTTCCCTTGATGGCCG |
|  | ADAMTS5 | Forward primer  Reverse primer | AAAGGGGAGAATCTGCCTGC  CCAAGATCCCCAGTTGCCAT |
|  | IL6 | Forward primer  Reverse primer | CCACCGGGAACGAAAGAGAA  GAGAAGGCAACTGGACCGAA |
|  | COl2A1 | Forward primer  Reverse primer | GCTCCTGCCGTTTCGCTG  ATTATACCTCTGCCCATCCTGC |
|  | ACAN | Forward primer  Reverse primer | CGTGTAAAAAGGGCACAGCC  GGAAGCTCTTCTCAGTGGGC |
|  | ACTB | Forward primer  Reverse primer | GTCATTCCAAATATGAGATGCGT  GCTATCACCTCCCCTGTGTG |
| Mouse | Adam19 | Forward primer  Reverse primer | TGTGTGATTGCGGACAGTGA  TAGCGGAGGGCTACCTTCTT |
|  | Cdkn2a | Forward primer  Reverse primer | CGAACTCGAGGAGAGCCATC  TACGTGAACGTTGCCCATCA |
|  | Cdkn1a | Forward primer  Reverse primer | CGGTGTCAGAGTCTAGGGGA  AGGATTGGACATGGTGCCTG |
|  | Lmnb1 | Forward primer  Reverse primer | AAGGCTCTCTACGAGACCGA  TTGAACTTGCCCAGCTCGAT |
|  | Mmp13 | Forward primer  Reverse primer | CTTCTGGCACACGCTTTTCC  ATGGGAAACATCAGGGCTCC |
|  | Adamts5 | Forward primer  Reverse primer | GAGCTAAGGGCACAGGCTAC  TGCCGTCACATCCAGTTCTC |
|  | Il6 | Forward primer  Reverse primer | TCCTACCCCAATTTCCAATGCT  TGGTCTTGGTCCTTAGCCAC |
|  | Col2a1 | Forward primer  Reverse primer | CATCTTGCCGCATCTGTGTG  TGCCCCTTTGGCCCTAATTT |
|  | Acan | Forward primer  Reverse primer | CCTGCTACTTCATCGACCCC  AGATGCTGTTGACTCGAACCT |
|  | Actb | Forward primer  Reverse primer | ACAGCAGTTGGTTGGAGCAA ACGCGACCATCCTCCTCTTA |

**Figures**

Figure S1


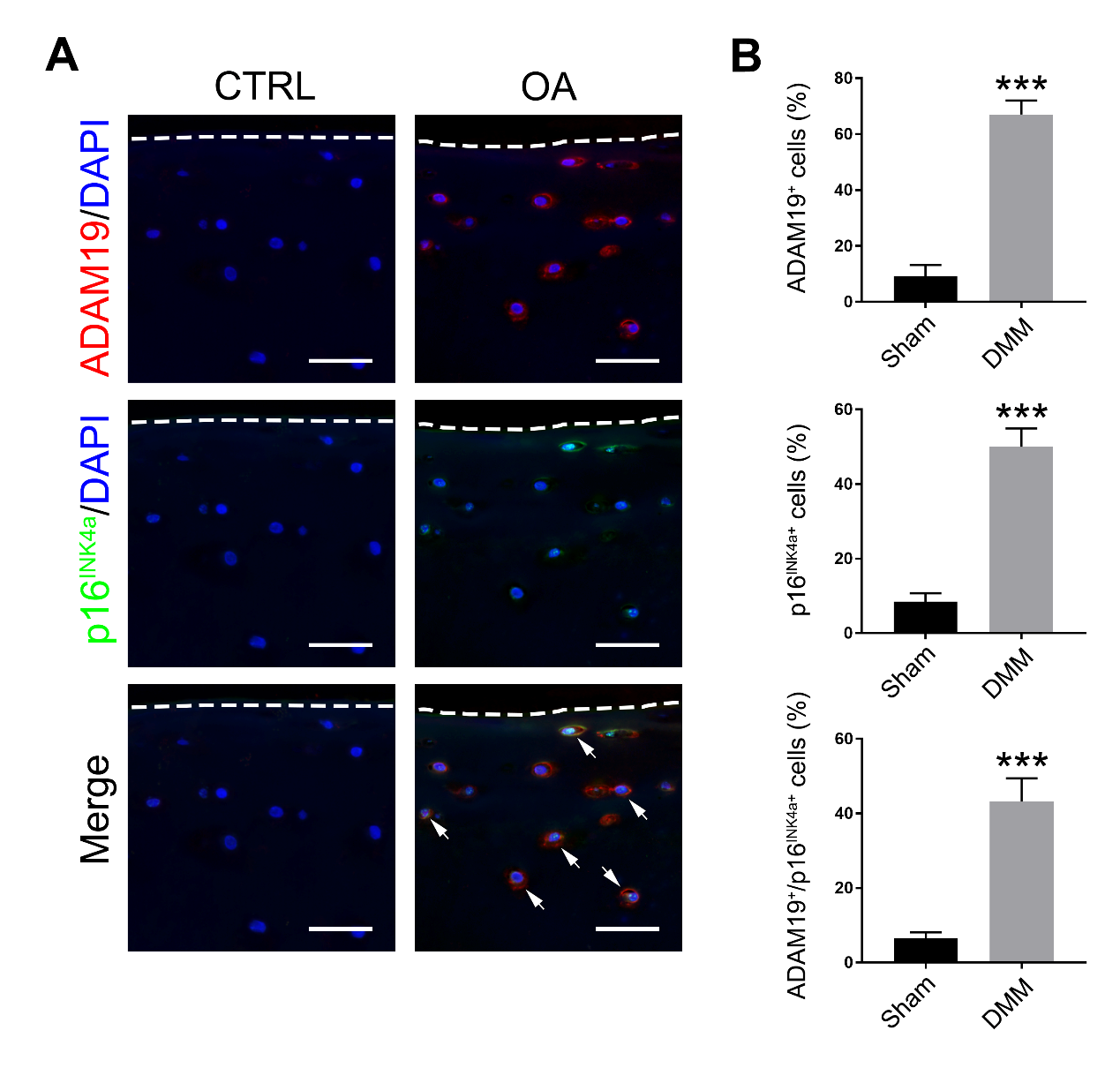


The expression of ADAM19 and p16^INK4a^ in OA patients. (A, B) Representative immunofluorescence images of ADAM19 (red) and p16^INK4a^ (green) in in human cartilage (A) and quantification of ADAM19, p16^INK4a^ and ADAM19/p16^INK4a^-double positive cells (B). n=3 donors for control and n=5 for OA. Scale bar, 50 μm. Dotted lines indicate the cartilage surface. Data are presented as means ± SD. ***p<0.001.

Figure S2


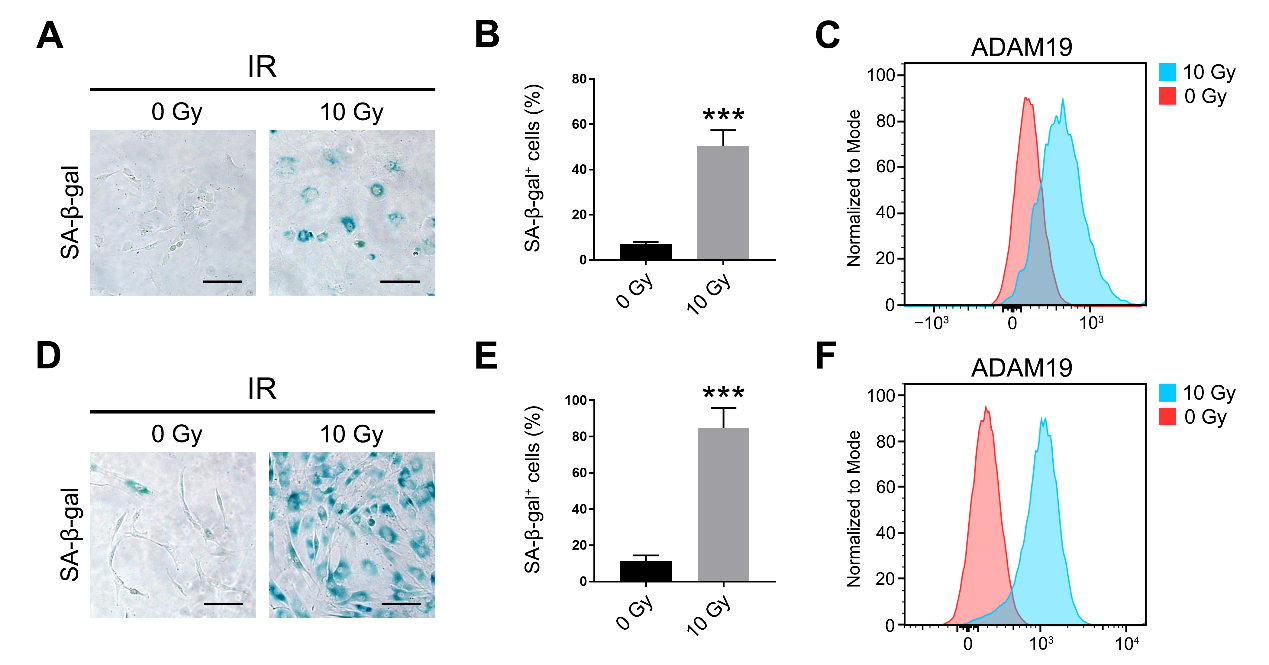


The expression of ADAM19 in mouse and human chondrocyte after IR exposure. (A, B) Representative images of SA-β-gal staining of mouse chondrocytes treated with 10 Gy X-ray irradiation (A) and quantification of SA-β-gal-positive cells (B). Scale bar, 30 μm. (C) Flow cytometry analysis of ADAM19 expression in IR-induced senescent mouse chondrocytes. (D, E) Representative images of SA-β-gal staining of human chondrocytes treated with 10 Gy X-ray irradiation (D) and quantification of SA-β-gal-positive cells (E). Scale bar, 30 μm. (F) Flow cytometry analysis of ADAM19 expression in IR-induced senescent human chondrocytes. Data are presented as means ± SD of at least 3 independent experiments. ***P < 0.001.

Figure S3


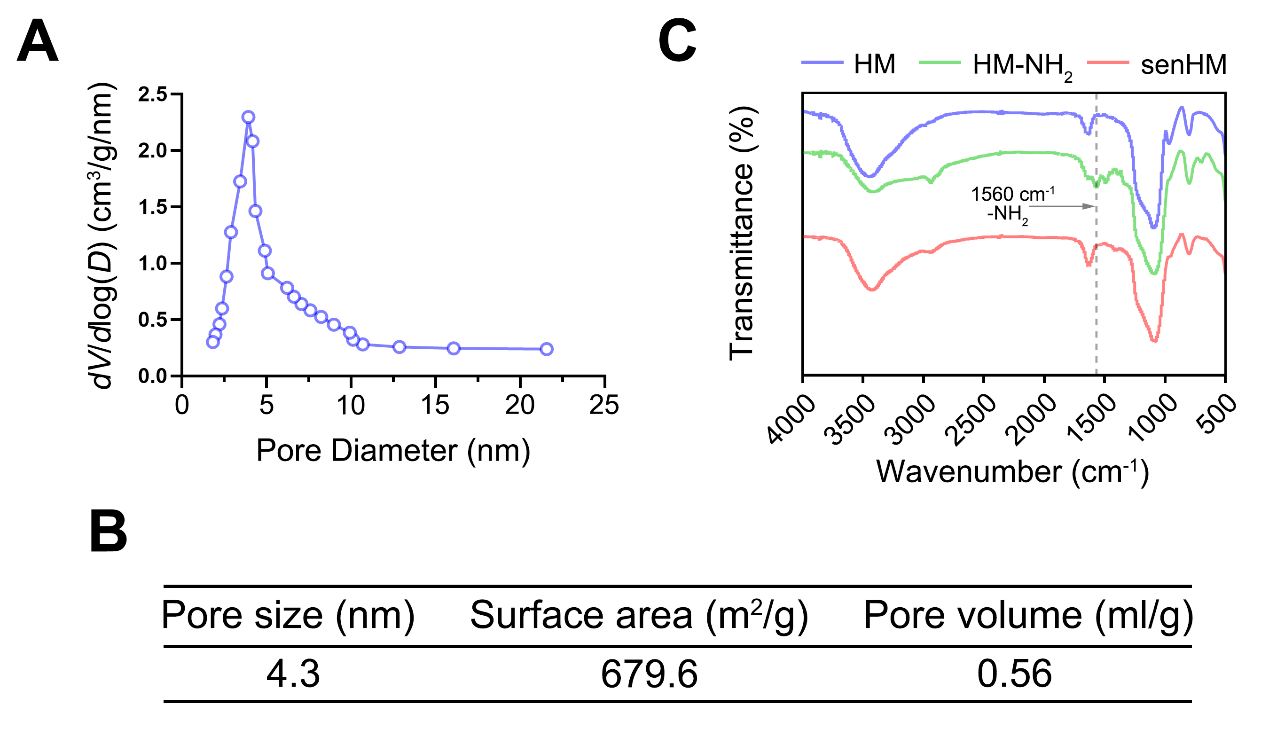


Characterization of HMSNs. (A, B) Pore size distribution (A) and pore size, surface area, and pore volumes of HMSNs (B). (C) FT-IR spectra of HMSNs (HM), amino-functionalized HM (HM-NH_2_), and senHMSNs (senHM).

Figure S4


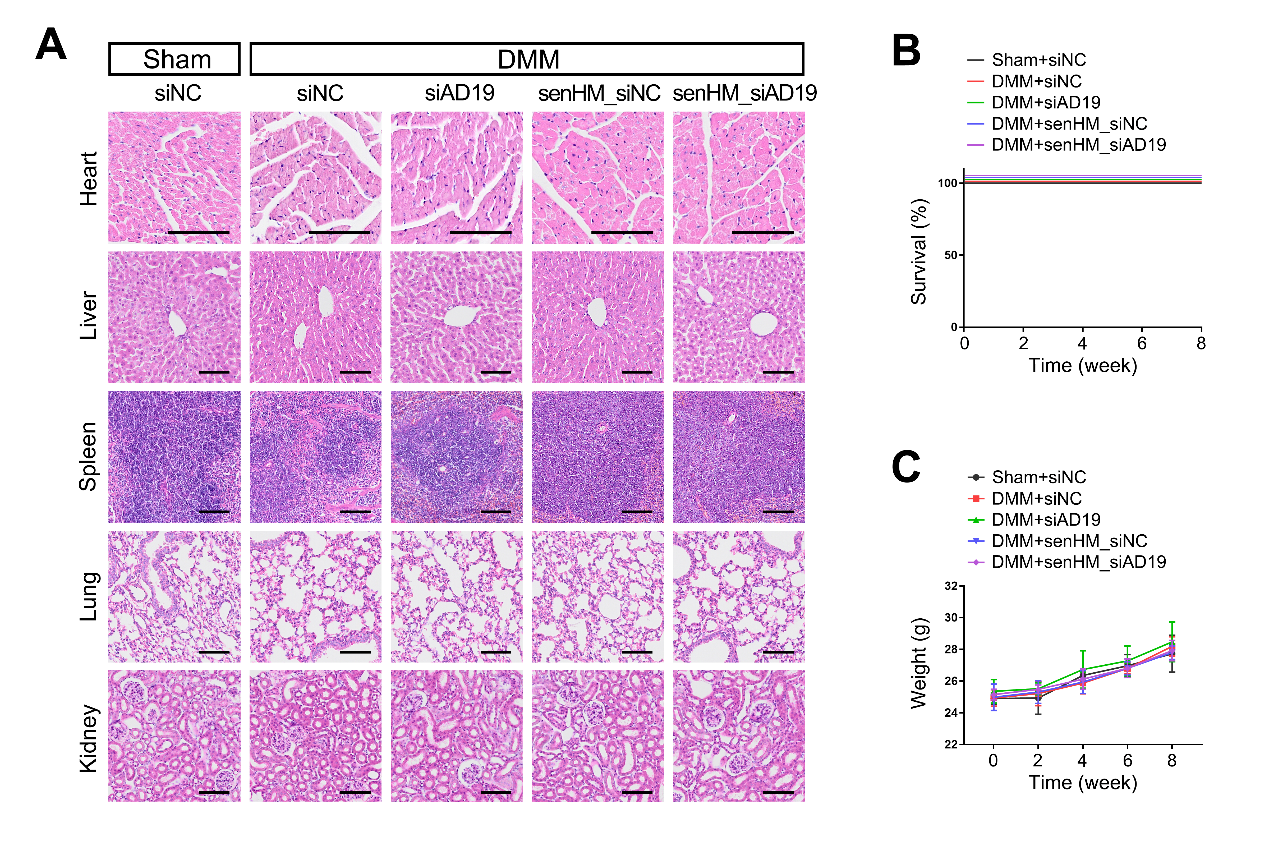


The biocompatibility of nanomaterials. (A) Representative H&E staining of major organs from the mice at the end of the treatment. Scale bar, 100 μm. (B) The survival of the treated mice. (C) The body weight of the mice during the whole treatment period. Sham+siNC, n=3; DMM+siNC and DMM+siAD19, n=5; DMM+senHM_siNC and DMM+senHM_siAD19, n=7. Data are presented as means ± SD.

Figure S5


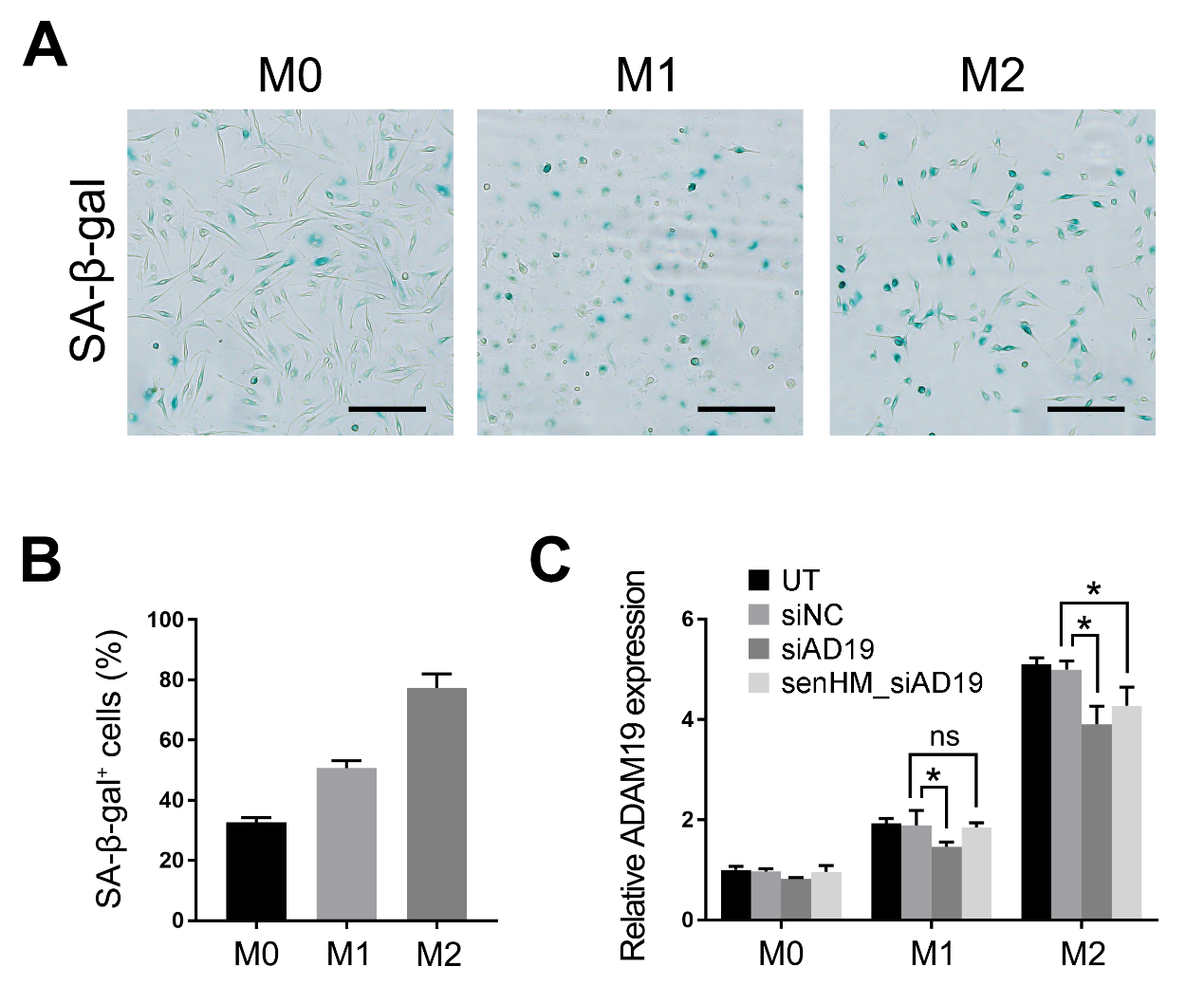


The effect of senHMSN_si-ADAM19 on macrophages under different polarization states. (A, B) Representative images of SA-β-gal staining of mouse bone marrow-derived macrophages (BMDMs) (A) and quantification of SA-β-gal-positive cells (B). Scale bar, 30 μm. (C) Transcriptional levels of target genes in mouse BMDMs. Mouse BMDMs were treated with 20 ng/ml M-CSF to induce M0, 100 ng/ml LPS+20 ng/ml IFN-γ to induce M1, and 20 ng/ml IL-4 to induce M2 polarization, respectively. Data are presented as means ± SD of at least 3 independent experiments. *P < 0.05, no significance (ns).

Figure S6

**
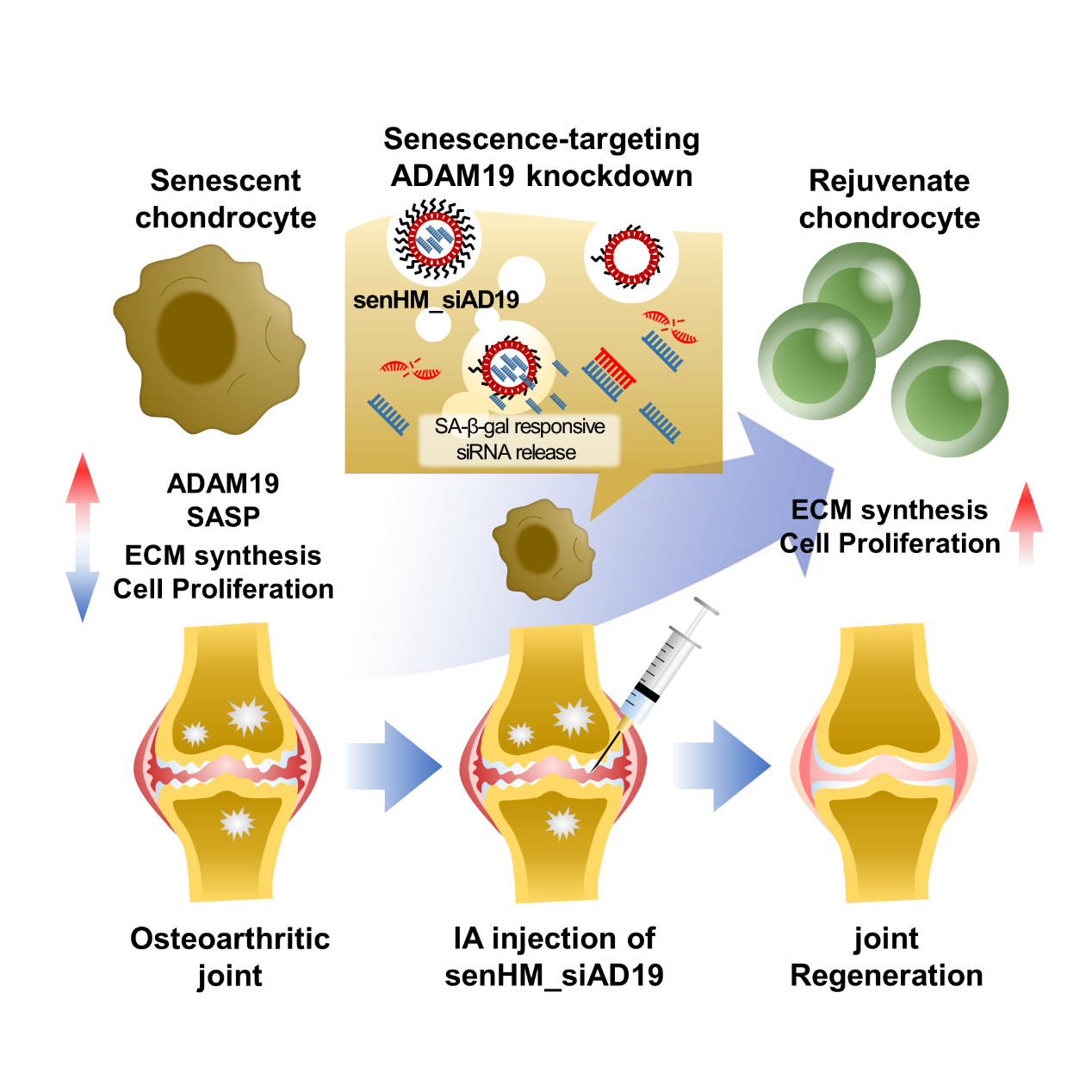
**

Schematic diagram of senHMSN_si-ADAM19 for OA treatment.
